# On-Road Driving Assessment in a Driving School Course and the Results of a Cognitive Function Test After Stroke in a Depopulated Rural Area in Japan: Case Series of Eight Patients

**DOI:** 10.7759/cureus.15293

**Published:** 2021-05-28

**Authors:** Keita Sakamaki, Shin Nishizawa, Masahito Katsuki, Shin Kawamura, Akihito Koh

**Affiliations:** 1 Department of Rehabilitation Medicine, Itoigawa General Hospital, Itoigawa, JPN; 2 Department of Neurosurgery, Itoigawa General Hospital, Itoigawa, JPN

**Keywords:** cognitive assessment, higher brain dysfunction, on-road driving assessment, rehabilitation, stroke, internal medicine in rural areas

## Abstract

Introduction

Returning to driving after a stroke is a step toward independence and improving quality of life. Cognitive function after stroke is one of the essential factors that affect driving ability, and on-road driving assessment in driving school courses is beginning to spread in Japan. We started on-road driving assessment in 2018, and we herein report eight patients who underwent on-road driving assessment in the last three years, presenting both off-road cognitive function tests and on-road driving assessment results.

Methods

Of the 320 consecutive stroke patients from 2018 to 2020, we retrospectively investigated the eight patients’ characteristics who underwent on-road driving assessment. We performed cognitive function tests, including behavioral inattention test (BIT), trail-making test, Wechsler Adult Intelligence Scale-III, and behavioral assessment of the dysexecutive syndrome. Patients who meet BIT > 35, at least three other subitem criteria, and no unevaluable subitems can undergo on-road driving assessment by a driving instructor. With the recommendation of the driving instructors, we comprehensively decided the permission to drive.

Results

All eight patients could return to driving after on-road driving assessment. Two patients could return to driving after nearly a year.

Conclusion

The patients did not meet all the cut-offs of the cognitive function test, but they were judged to return to driving by driving instructors. We finally permitted all eight patients to drive. On-road driving assessment in the driving school course might be helpful for determining the permission to return to driving.

## Introduction

Returning to driving after a stroke is a step toward independence and improving quality of life. In Japan, stroke patients were almost uniformly disqualified from driving licenses before 2002. From 2002, the Road Traffic Law was amended to allow some stroke patients to drive according to their symptoms. If the symptoms are expected to improve within six months, the license will be put on hold and then the tests repeated. If the symptoms improve within six months, the patients are allowed to drive. After six months without improvement, the license will expire. However, if the symptoms improve within three years, even if the license was once revoked, it can be reacquired without any tests using a medical certificate that the patients' cognitive function improved [1]. Nevertheless, the criteria of cognitive function improvement are not clearly defined in Japan.

Cognitive function after stroke is one of the essential factors that affect the driving ability [2], and many off-road (paper and simulator) tests were used to assess the cognitive functions as screening [3-6]. However, the golden standard for driving is the “on-road” test [4,7]. On-road driving assessment in the driving school course is beginning to spread in Japan. In 2017, 210 hospitals collaborated with driving schools to provide rehabilitation for returning to driving [8], and its efficacy and cost were under discussion [9-10].

We work in a rural area in Japan, with the aging population constituting 40.3% and with an insufficient public transportation system. We should prevent traffic accidents by patients after stroke but also maintain their quality of life. We started on-road driving assessment in 2018. We herein report eight patients who underwent on-road driving assessment in the last three years, presenting both off-road cognitive function tests and on-road driving assessment results in the driving school course.

## Materials and methods

Study population

We retrospectively investigated 320 consecutive stroke patients who were admitted to the department of neurosurgery in our hospital between 2018 and 2020. We treated them according to the Japanese Guidelines for the Management of Stroke 2015 [11]. Physical, occupational, and speech therapies were started as soon as possible, and cognitive function assessment was performed parallel with rehabilitation. From the 320 stroke patients, we found eight patients who underwent on-road driving assessment in the driving school course and studied their characteristics.

Our hospital’s research ethics committee approved the study, and we gained written informed consent for this study from all of the patients, the legally authorized representative of the patients, or the next of kin of the deceased patients. All methods were carried out under relevant guidelines and regulations (Declaration of Helsinki). All personal patient information was deleted from the database for this study to protect patient privacy.

Driving assessment and cognitive assessment

We performed a rehabilitation program for returning to driving according to the proposal of Niigata Rehabilitation Hospital [9]. First, patients who wanted to drive must meet all these conditions: 1) modified Rankin Scale 0-1, 2) able to go out alone, 3) no severe motor dysfunction for driving, 4) aware of the disease and no dangerous behavior, 5) able to communicate at the level of daily conversation, 6) able to manage finances and handle problems, and 7) able to plan and remember daily activities. Second, we performed a cognitive function assessment composed of four tests, including behavioral inattention test (BIT), trail-making test (TMT)-A and B, Wechsler Adult Intelligence Scale-III (WAIS-III), and behavioral assessment of the dysexecutive syndrome (BADS). The cut-off values are shown in Table 1.

**Table 1:** Cognitive function assessment performed in our hospital *Patients who meet the behavioral inattention test, at least three other items, and no unevaluable items can undergo the on-road driving assessment by a driving instructor.

Order	Neuropsychological examination	Cut-off values for each item*
1	Behavioral inattention test	≧35
2	Trail making test–A and B	A; within 65 sec
		B; within 192 sec
3	Wechsler Adult Intelligence Scale-III	Digit Symbol; ≧7
		Symbol Search; ≧7
		Arithmetics; ≧7
		Digit Span; ≧7
		Letter-Number Sequences; ≧7
4	Behavioral assessment of the dysexecutive syndrome	Zoo map; ≧2
		Modified six elements ≧3

We permitted the patients who met all the criteria to drive without on-road driving assessment. However, if there was even one criterion that did not meet the cut-off, we did not permit them to drive. Such patients were considered for on-road driving assessment. Patients who met the behavioral inattention test, at least three other items, and no unevaluable items could undergo the on-road driving assessment in the driving school course, not in the public road nor real traffic, evaluated by a driving instructor. If patients could not meet the standards for on-road assessment described above, we re-evaluated the patients at the outpatient after some months as they wanted. Once the patients reached the standards, they could undergo the on-road driving assessment. Finally, the driving instructor evaluated the patient using the scoresheet with 20 items evaluated by five-point ordinal scales (Table 2) [9]. Doctors judged whether they could return to driving or not based on the comprehensive results. If patients could not give up returning to driving, we recommend that they consult the police station. The police officers let the patients consult us to make the medical certificate, and usually, the police officers make the patients stop driving.

**Table 2:** On-road driving assessment scoresheet for the driving instructor The driving instructor evaluated the patient using the scoresheet with 20 items evaluated by five-point ordinal scales.

Items		Evaluation (1-5 points)	Items		Evaluation (1-5 points)
Posture and starting procedure	Driving posture		Law compliance and essential operation	Traffic signal compliance	
	Checking the mirrors			Changing lanes	
	Starting procedure			Left turn	
Narrow road	S junction			Right turn	
	Cranking			Speed control	
Attention and safety	Parking in reverse			Going around a curve	
	Intersection			Keep left	
	Temporary stop			Braking operation	
	Behavior after advice		Motor function	Handle operation	
	Sideways passage of obstacles			Total evaluation	
Comments from the driving instructor					

Regarding patients who did not wish to drive, who were physically unable to drive due to comorbidities like fractures and wish to stop driving, or were requested not to drive by caregiving family members, this rehabilitation program for driving was not performed.

## Results

Of the 320 stroke patients (143 women and 177 men, mean ± standard deviation (SD) of age 76±11.7 y.o.), 81 patients had intracerebral hemorrhages, 28 patients had subarachnoid hemorrhages, and 211 patients had ischemic cerebrovascular diseases. Thirty-seven patients could be discharged home with mRS 0-1 (15 women and 22 men, mean ± sd of age 71±11.6 y.o.). Among the 37 patients with mRS 0-1, 16 patients directly returned to driving, seven patients had not usually driven, four patients did not wish to drive, and two patients could not return to driving due to mild dementia and were requested not to drive by caregiving family members. Therefore, eight patients did not meet some of the cut-offs and underwent an on-road driving assessment.

Table 3 summarizes the characteristics of the eight patients (2 women and 6 men, mean ± SD of age 60.3±13.0 y.o.) who underwent on-road driving assessment. Four patients had cerebral infarctions, three intracerebral hemorrhages, and one subarachnoid hemorrhage. The mean Glasgow Coma Scale score on admission was 12.8±1.8.

**Table 3:** Patient characteristics of the eight patients who underwent on-road driving assessment ATBI: atherothrombotic brain infarction, BADS: behavioral assessment of the dysexecutive syndrome, BIT: behavioral inattention test, CE: cardiogenic embolism, iv rt-PA: intravenous recombinant tissue-plasminogen activator, TMT: trail-making test, WAIS-III: Wechsler Adult Intelligence Scale-III, *; better than the cut-off, †: Patients who meet the behavioral inattention test, at least three other items, and no unevaluable items can undergo the on-road driving assessment by a driving instructor.

Patient No.	Pt. 1	Pt. 2	Pt. 3	Pt. 4	Pt. 5	Pt.6	Pt.7	Pt. 8
Age, Sex	41 M	46 W	51 M	61 M	66 W	68 M	71 M	78 M
Stroke type (treatment)	Basilar occlusion due to ATBI (iv rt-PA, thrombectomy)	Subarachnoid hemorrhage due to Rt A4 dissection (trapping)	Lt thalamic hemorrhage (medical treatment)	Lt putaminal hemorrhage (medical treatment)	ATBI, Rt M2 superior trunk occlusion (medical treatment)	Lt putaminal hemorrhage (medical treatment)	CE due to atrial fibrillation (medical treatment)	Watershed infarction due to lt neck internal carotid artery stenosis (iv-rt-PA, carotid endoarterectomy)
Glasgow Coma Scale score on admission	10	14	14	14	11	15	12	12
E	3	4	4	4	4	4	4	3
V	1	4	4	4	1	5	3	3
M	6	6	6	6	6	6	5	6
Cognitive function assessment during hospitalization								
BIT	37*	37*	39*	38*	37*	40*	38*	38*
TMT-A	88	95	99	125	96	94	180	126
TMT-B	122*	104*	117*	164*	149*	163*	381	Unevaluable
WAIS-III Digit Symbol	8*	3	7*	6	9*	11*	7*	10*
WAIS-III Symbol Search	5	4	5	9*	6	9*	7*	6
WAIS-III Arithmetics	7*	7*	5	9*	6	15*	5	5
WAIS-III Digit Span	8*	5	4	15*	6	7*	4	6
WAIS-III Letter-Number Sequences	5	9*	6	7*	5	10*	5	6
BADS, zoo map	Unevaluable	2*	3*	2*	5*	2*	0	1
BADS, modified six elements	3*	1	1	2	4*	3*	Unevaluable	4*
First evaluation for trying on-road test†	Unable	Able	Unable	Able	Able	Able	Unable	Unable
Cognitive function assessment at outpatient								
Days from the onset	93		63				272	55
BIT	40*		40*				38*	40*
TMT-A	81		71				168	93
TMT-B	73*		161*				219	146*
WAIS-III Digit Symbol	8*		8*				8*	12*
WAIS-III Symbol Search	8*		4				7*	10*
WAIS-III Arithmetics	6		8*				5	5
WAIS-III Digit Span	11*		5				3	5
WAIS-III Letter-Number Sequences	8*		8*				5	7*
BADS, zoo map	2*		3*				0	4*
BADS, modified six elements	3*		4*				4*	4*
Second evaluation for trying on-road test†	Able		Able				Able	Able
On-road driving assessment by driving instructor								
Days from the onset	136	61	109	44	313	43	300	128
Driving posture	4	3	4	4	4	4	4	4
Checking the mirrors	5	4	5	5	4	4	4	4
Starting procedure	4	4	4	4	5	4	4	4
S-shape junction	4	4	5	3	5	3	4	5
Cranking	3	5	5	4	5	3	4	5
Parking in reverse	4	5	5	4	5	5	4	5
Intersection	4	5	5	5	4	4	5	4
Temporary stop	3	4	5	4	5	5	5	4
Behavior after advice	4	5	5	5	5	4	5	5
Sideways passage of obstacles	4	4	3	4	5	4	3	5
Traffic signal compliance	5	5	5	5	5	5	5	5
Changing lanes	3	3	3	4	4	4	4	3
Left turn	3	4	3	4	4	2	4	3
Right turn	4	3	5	4	4	4	4	3
Speed control	3	4	5	4	5	5	3	5
Going around a curve	3	4	5	3	4	4	4	4
Keep left	3	3	5	4	5	4	4	4
Braking operation	3	4	5	5	5	4	4	4
Handle operation	4	3	5	5	4	3	4	3
Total evaluation	4	4	4	4	5	3	4	4

For the first cognitive function assessment during the hospitalization, four of the eight patients met the criteria and underwent an on-road driving assessment. The rest of the four patients underwent the secondary cognitive function assessment at the outpatient. The days from the onset to the secondary evaluation ranged from 55 to 272 days. In the on-road driving assessment in the driving school course, all eight patients were judged to return to driving by driving instructors, although some of the cognitive function tests did not meet the cut-off. The days from the onset to the on-road driving assessment ranged from 43 to 313 days. After a comprehensive evaluation, we finally permitted all eight patients to drive. In the follow-up period ranging from six to 36 months after the on-road driving assessment, there have been no traffic accidents caused by the patients.

## Discussion

We herein reported eight stroke patients who underwent on-road driving assessments. Some patients did not meet all the cut-offs of the cognitive function tests. However, they were judged to return to driving in the on-road driving assessment by driving instructors, so we finally permitted all eight patients to drive after a comprehensive evaluation. Our report suggests that some stroke patients can drive, which cannot be predicted by off-road cognitive function tests alone, and that an on-road driving assessment on the driving school course is useful.

Off-road and on-road driving assessment in Japan

People with stroke have variable deficits, which can influence their driving ability, including reduced visual scanning, attention, information-processing speed, and visuospatial skills. Therefore, cognitive function assessment (off-road) and on-road driving assessment are important. Off-road and on-road driving assessments are now widespread in Japan since 2002 when the Road Traffic Law was amended. However, reports on the driving assessment remain few. This is because each academic association or hospital conducts its own assessment, so there are no consistent driving assessment procedures. Also, “on-road driving assessment” in Japan means a driving test “in the driving school course.” This is another problem, as we cannot predict the patients’ performance in real-time traffic.

A famous off-road cognitive function test, Stroke Drivers’ Screening Assessment Japanese Version (J-SDSA) is widely used, but its accuracy for on-road performance is about 70% [12]. Therefore, SDSA alone cannot determine if a person can drive; it is only a screening. The Japan Society for Higher Brain Dysfunction proposes “Japan Society for Higher Brain Dysfunction’s driving assessment (draft)" [5]. This proposal focuses on several cognitive function tests, including Mini-Mental State Exam, Hasegawa Dementia Scale-Revised, BIT, TMT, WAIS-III, Rey complex figure test, Kohs block design test, Frontal Assessment Battery, Wisconsin Card Sorting Test, and BADS. However, the proposal does not describe on-road driving assessment in detail, and the burden on the patients and medical staff to perform all the cognitive function tests is heavy. The patients’ concentration may also be interrupted during these many tests. Therefore, each hospital modifies the test compositions and the cut-off values [6,9,13-15]. The composition and cut-off values of these cognitive function tests should be continuously discussed.

According to on-road driving assessment, the Japanese Association of Occupational Therapists [16] and the Japan Federation of Authorized Drivers School Associations [10] cooperatively performed research on off-road cognitive functions and on-road driving tests on the driving school course. The number of hospitals and driving schools where both off- and on-road driving assessments are performed increased to over 200 in 2017 [8]. However, there are no definite cut-off values for on-road driving assessments on the patients' driving performance. Hence, doctors decided whether patients can return to driving based on the qualitative and quantitative reports from the rehabilitation therapists and driving instructors. Besides, some hospitals with limited medical resources have to decide based on off-road tests alone without on-road tests [6]. There are also some problems with on-road driving assessments for driving schools: lack of human resources, clear standards, and medical knowledge, as well as disruption to everyday work for young people to get their drivers’ licenses.

Some of the on-road driving assessments worldwide are useful [7]: the Washington University Road Test (WURT), Rhode Island Road Test (RIRT), Test Ride for Investigating Practical fitness-to-drive (TRIP), and Performance Analysis of Driving Ability (P-drive). In the WURT, the patients start driving from the parking lot, closed course, and then on an urban open road with two to six lanes with a 9.6-km course. Two well-trained driving instructors judged permission using 36 items with a three-point ordinal scale [17]. RIRT [18] is similar to WURT. TRIP [19] contains 13 evaluative items and 49 subitems on a four-point ordinal scale using clear-cut guidelines for each of the 49 subitems after simulator and on-road driving. P-drive consists of an assessment tool with 27 items by a four-point ordinal scale related to driving on-road with a detailed and structured manual [20].

The most different aspect from the Japanese on-road driving assessment is that these on-road tests abroad are performed on a “real traffic road.” This is because the Road Traffic Law in Japan temporarily revokes the drivers’ license when the cognitive disorder remains over six months. Even if within six months, the license will be put on hold and patients cannot drive on public roads. Of our eight patients who could return to driving, two underwent driving assessment after over six months from the stroke onset and returned to driving. Therefore, although on-road driving assessment in the public road cannot be performed in Japan, those in the driving school course might be useful and some stroke patients could return to driving after over 6 months. The necessity of on-road driving assessment “in real traffic” needs further discussion. In conclusion, although it is costly, it is important to consider the best way for each stroke patient, rather than having him/her give up their driver’s license just because of the bad results of off-road cognitive function tests alone.

Limitations

First, the sample size of the current study is small, and we should continue to study larger samples and perform statistical analysis to reveal which patients will be able to drive with/without on-road driving assessment. Second, we should have investigated the patients’ and their families’ psychological status and physical burden caused by these driving assessments or driving license suspension in this rural area. Third, it is unclear whether cognitive function deteriorated due to stroke or if the patients already had some degree of cognitive impairment due to other diseases such as dementia, etc.

## Conclusions

We herein reported on eight stroke patients who underwent on-road driving assessments on a driving school course. The patients did not meet all the cut-offs of the cognitive function test, but they were judged to return to driving by driving instructors. Therefore, we finally permitted all eight patients to drive, and all patients have caused no traffic accidents. On-road driving assessment on the driving school course might help determine the permission to return to driving.
